# The Stressogenic Impact of Bacterial Secretomes Is Modulated by the Size of the Milk Fat Globule Used as a Substrate

**DOI:** 10.3390/foods13152429

**Published:** 2024-08-01

**Authors:** Noam Tzirkel-Hancock, Chen Raz, Lior Sharabi, Nurit Argov-Argaman

**Affiliations:** Department of Animal Science, The Robert H Smith Faculty of Agriculture, Food and Environment, The Hebrew University of Jerusalem, Rehovot 7610001, Israel; noam.tzirkel@mail.huji.ac.il (N.T.-H.);

**Keywords:** mammary epithelial cells, stress, lipid droplets, postiobtics

## Abstract

Milk fat globules (MFGs) are produced by mammary epithelial cells (MECs) and originate from intracellular lipid droplets with a wide size distribution. In the mammary gland and milk, bacteria can thrive on MFGs. Herein, we aimed to investigate whether the response of MECs to the bacterial secretome is dependent on the MFG size used as a substrate for the bacteria, and whether the response differs between pathogenic and commensal bacteria. We used secretomes from both *Bacillus subtilis* and *E. coli*. Proinflammatory gene expression in MECs was elevated by the bacteria secretomes from both bacteria sources, while higher expression was found in cells exposed to the secretome of bacteria grown on large MFGs. The secretome of *B. subtilis* reduced lipid droplet size in MECs. When the secretome originated from *E. coli*, lipid droplet size in MEC cytoplasm was elevated with a stronger response to the secretome from bacteria grown on large compared with small MFGs. These results indicate that MEC response to bacterial output is modulated by bacteria type and the size of MFGs used by the bacteria, which can modulate the stress response of the milk-producing cells, their lipid output, and consequently milk quality.

## 1. Introduction

Milk fat is secreted in a structure termed milk fat globule (MFG), which consists of a triglyceride core enveloped by three layers of polar lipids, proteins, and glycoconjugates, together termed the milk fat globule membrane (MFGM). MFGs are secreted into milk in a wide range of sizes, a property that is well conserved throughout the evolution of mammals as has been recorded in ruminants [1], pigs [2], mice [3], and humans [4,5]. As a conserved trait, it is hypothesized that it provides a benefit either to the lactating mother or her infant. However, this trait is currently understudied, and there is no clear understanding of how this size diversity is maintained or its importance.

The biosynthesis pathway of milk lipids is executed by a set of biochemical sequential steps that are common to many other lipogenic tissues, including adipose and liver. The process starts between the leaflets of the endoplasmic reticulum (ER), where the esterification of fatty acids to glycerol forms triglyceride molecules, which form a hydrophobic droplet [6]. The fatty acid source can be pre-formed fatty acids derived from the blood, or de novo synthesized fatty acids produced by acetyl CoA carboxylase (ACC) and fatty acid synthase (FASN). Once reaching a certain size, each droplet is released into the cytoplasm, covered with one layer of polar lipids derived from the ER. This structure is called a micro-lipid droplet (MLD). From their site of origin, the MLDs migrate to the apical pole of the cell, acquiring cytoplasmic proteins that dock onto the surface of the droplet and the cytoplasmic face of the apical membrane, which together facilitate apocrine secretion from the cell into the alveoli lumen [7]. During synthesis, migration toward the apical membrane, and secretion as an MFG, several biochemical, mechanical, and physical processes can modulate the size of the lipid droplet. For example, enzymes that synthesize (i.e., diacylglycerol acyl transferase, DGAT) or hydrolyze (adipose triglyceride lipase, ATGL) triglycerides directly on the surface of the droplet can change its size [8,9], and adipophilin (ADPH), which regulates the adherence of lipases onto the surface of the lipid droplet, can contribute to the lipid droplet size modulation [10] and, consequently, the secreted MFG. These enzymes are usually regulated by metabolic hormones and signals and the energy level of cells, often sensed by AMP levels and regulatory factors like sirt1 [11,12]. Another metabolic factor that can change the lipid droplet size is the availability of pre-formed fatty acids in the form of non-esterified fatty acids (NEFA) or lipoproteins (specifically very low-density lipoproteins) [13]. Their relative abundance in the blood is also a consequence of energy balance or time postprandial. Energy levels in the cell and mitochondria activity can also modulate the lipid droplet size through alteration in phospholipid composition, and modulation of fusion events that occur between adjacent cytoplasmic lipid droplets [14].

Taken together, since the majority of the aforementioned processes are associated with the mitochondria and energy status, changes in the oxidative status of the cells primarily due to external stressors are hypothesized to impact the size and number of intracellular lipid droplets.

The implication of stress on the lipid content in the cells and the number and size of lipid droplets seems to be tissue-specific. Tissues that accumulate fat may respond differently to stress conditions compared to tissues with lipid processing and secretion roles. In the liver, for example, pathologies such as non-alcoholic fatty liver disease cause an increase in lipogenesis and lipid droplet production, leading to their accumulation and potential fusion and size increase [15]. Also, the greater antioxidant capacity of adipose tissue relating to the oxidative status of the cell has been found to be associated with lipid droplet storage capacity, with larger lipid droplets having a greater antioxidant capacity [16]. In skeletal muscle, energy stress is manifested in induced mitochondria lipid droplet co-localization and reduced size of cytoplasmic lipid droplets [17].

Another factor that can highly affect lipid droplet size is the availability of envelope material. Changes in the production capacity of phospholipids such as phosphatidylcholine (PC) can occur due to oxidative stress, as PC is involved in antioxidative processes [18]. Changes in envelope material can lead to an increase in lipid droplet size, as was demonstrated in drosophila S2 cells [19]. However, in the mammary gland, it is not clear if the proinflammatory response to external stressors elicits the same modulation of lipid droplet size and number.

After the formation of the cytoplasmic lipid droplet, the lipogenesis in the mammary gland diverges from the promiscuous process in other lipogenic tissues (i.e., liver and adipose), resulting in the acquisition of an additional bilayer surrounding the initial micelle. The fact that eventually MFGs differ significantly from intracellular lipid droplets used for energy storage or excreted lipoproteins is probably attributed to the fact that MFGs evolved as food. Moreover, unlike lipid droplets derived from adipose tissue and the liver, MFGs are secreted into mammary gland cavities. These cavities are exposed to the exterior of the mammary gland and are often populated with a microbiome consisting of environmental and skin bacteria [20]. It was previously shown that bacteria strains that are usually found in the dairy farm, dairy plants, and skin of the udder are found within the mammary gland, and thus exposed to milk components. Specifically, *Bacillus subtilis* and *E. coli* were shown to thrive on milk [20,21], with *Bacillus subtilis* exhibiting different growth trajectories depending on the size of MFGs used as a substrate [22], resulting in the production of a compositionally distinguished secretome (i.e., postbiotics, PB). Therefore, the mammary gland parenchyma, and especially the epithelial lining of the milk collecting ducts and alveoli, are exposed to metabolites released by the bacteria, which can induce a stress response due to toxins or other metabolites.

In the present study, we assessed the possibility that the size and number of intracellular lipid droplets can be used as a proxy for determining a stress response in MECs. Moreover, these properties were used to assess the stress and metabolic response to bacterial secretomes, which were modulated by the size of the MFGs used as a substrate. We found that MECs modulate the size and number of lipid droplets in response to extreme stressors, even if the oxidative status of the cell is not equally affected by the stressor. Moreover, we found that the proinflammatory response to the bacterial secretome is affected by the size of MFGs used as a substrate, as was reflected by both changes in the intracellular lipid droplet phenotype and proinflammatory cytokine gene expression. To allow a robust phenotyping of lipid droplets in the cells, we wrote a script that performed automatic image analysis and provided information on the number of lipid droplets per cell and their average diameter. We used this mechanism to shed light on how the stress response in MECs may impact their lipid output, and potentially change milk quality.

## 2. Materials and Methods

### 2.1. Primary Culture

In all experiments, bovine mammary epithelial cells isolated from Holstein cows served as the primary culture. The isolation procedure was previously established in our laboratory [23]. The study protocols adhered to the regulations set forth by the Israeli Ministry of Health and were supervised by the Department for Control of Animal Products, State of Israel Ministry of Agriculture Rural Development Veterinary Services and Animal Health. Udder tissue was collected from lactating Holstein in a commercial slaughterhouse and immediately submerged in a growth medium. The growth medium contained DMEM/F12, L-Glutamine (292 mg/mL), Penicillin (100 μg/mL), Streptomycin (100 μg/mL), Amphotericin (0.25 μg/mL), 10% FBS, Insulin (1 μg/mL), and hydrocortisone (0.2 μg/mL). Upon arrival at the laboratory, 12.5 g of the udder tissue pieces were digested in 100 mL of growth medium supplemented with collagenase (1 mg/mL) and hyaluronidase (1 mg/mL) in a 500 mL Erlenmeyer flask. The flask was shaken at 100 rpm for 4 h at 37 °C. After the incubation period, we filtered the suspension through a Nitex mesh (250 μm), and the filtrate was centrifuged at 350× *g* for 5 min. The supernatant containing the fat and enzymes was discarded, and the cell sediment was treated with trypsin–EDTA solution C. Subsequently, the cells were washed with growth medium, treated with 0.04% (*w*/*v*) DNase, and filtered through a 100 μm filter. A growth medium was added to the cells in order to reach the desired volume of 50 mL. Roughly 500,000 cells were then plated on 100 mm plated in 10 mL of growth medium. The medium was changed after 24 h and henceforth after every 48 h. The cells were grown until they reached 100% confluence, after which they were dispersed using 0.05% (*w*/*v*) trypsin and transferred to new plates.

### 2.2. Materials

DMEM/F12, fetal bovine serum (FBS), penicillin, streptomycin, amphotericin B, L-glutamine solution, and trypsin–EDTA solution C were purchased from Biological Industries (Beit Haemek, Israel). Bovine insulin, hydrocortisone, ovine prolactin, bovine serum albumin (BSA) solution, hyaluronidase, DNase I, heparin, sodium azide, sodium fluoride, and DZA were purchased from Sigma Aldrich Israel Ltd. (Rehovot, Israel). Collagenase type II was purchased from Worthington Biochemical Corporation (Lakewood, NJ, USA).

### 2.3. Bacterial Secretome Collection

For experiments with postbiotics, we used wild-type cells of *B. subtilis* NCIB 3610 and wild type *E. coli* p4 isolated from raw milk that was collected from Holstein Israeli dairy cows from a commercial herd (Volcani dairy farm, Rishon Letzion, Israel). Bacteria were grown on Lysogeny Broth (LB;10 g of tryptone, 5 g yeast extract and 5 g of NaCl per liter) or LB solidified with 1.5% agar (Difco, Le Pont de Clainx, France), mixed with small or large MFGs. Small and large MFG were separated from raw milk, as previously described [22], and consisted of MFGs with an average diameter of 2.3 ± 0.3 µm (i.e., small MFGs) or 7 ± 0.1 µm (i.e., large MFGs). After 24 h of incubation of bacteria with small or large MFGs, supernatant was collected and filtered through a 0.22 µm filter to remove bacteria. In addition, following incubation the bacteria number was determined by CFU. Postbiotics metabolite concentration was adjusted according to the CFU results, to reflect the amount of metabolites released by the same number of bacteria cells.

### 2.4. Experimental Design

Primary MEC were plated at 150,000 cells per 60 mm plastic dish for RNA extraction or at 50,000 cells per well in 6-well plates on glass coverslips for Nile red staining. Growth medium was replaced every 48 h during incubation. Once cells grew to 80% confluence, the medium was replaced with DMEM/F12 without serum, containing 0.15% (*w*/*v*) fatty acids–free BSA and insulin (1 μg/mL), hydrocortisone (0.5 μg/mL), and prolactin (1 μg/mL) for 48 h to induce milk lipid and protein synthesis. Treatment medium was added, consisting of DMEM/F12 supplemented with 0.5% (*w*/*v*) free fatty acids–free BSA, insulin (1 μg/mL), hydrocortisone (0.5 μg/mL), and prolactin (1 μg/mL), as well as 0.2 μg/mL or 1 μg/mL of LPS, 0.5 μM of H_2_O_2_, or 20% postbiotics from *E. coli* or *B. Subtilis* grown on small or large MFGs. For Nile red staining, 360 μM of Oleic Acid (18:1) was added to the treatment medium.

### 2.5. Lipid Droplets Staining

Cells were plated and grown on glass coverslips. The coverslips were rinsed three times with PBS and fixed with 1 mL of 4% paraformaldehyde for 20 min at room temperature. The coverslips were then rinsed with PBS four times and stained with 1 mL of Nile red (200 nM diluted in PBS, Sigma, St. Louis, MO, USA) for 15 min. Next, the coverslips were rinsed three times with PBS and stained with 1 mL DAPI (Sigma, St. Louis, MO, USA) for 5 min. Finally, the coverslips were rinsed four times with PBS and mounted with a fluorescent mounting medium (Dako, North America Inc., Carpinteria, CA, USA). Cells were visualized through an Echo inverted fluorescence microscope (Bico, San Diego, CA, USA).

### 2.6. Fluorescence Microscopy and Lipid Droplet Size Measurements

Lipid droplets were analyzed for size and number in order to capture the phenotype of MECs under stress and to assess the stress induced by postbiotics. In order to increase the efficiency of image analyses, we wrote a script that aimed to analyze a large number of figures in a shorter period of time. In addition, the automated process aimed to overcome the limitation of image adjustment in order to capture both large and small lipid droplets in the same cell: to capture all lipid droplet sizes, images must be adjusted in a way that will either impair our ability to quantify large lipid droplets or the capacity to determine the size of small lipid droplets. Therefore, lipid droplets were measured by either manual or automatic analysis. For the manual analysis, lipid droplet diameter was measured using ImageJ software (version 1.48, NIH, Bethesda, MD, USA). Values represent the mean size of all droplets in the cells. For the automatic analysis, images of MEC stained with Nile Red were run through a Python script for analysis. Each image was loaded, converted to greyscale, and had its contrast adjusted for better analysis, using contrast-limited adaptive histogram equalization, with a 0.03 clip limit. Circular droplets were detected using the ‘skimage’ Python library (https://github.com/scikit-image/scikit-image (accessed on 9 September 2023)). Using the OpenCV Python library (https://github.com/opencv/opencv (accessed on 9 September 2023)), the coordinates of each previously detected droplet were added in white onto a black background and saved as a separate image. Utilizing the same library, droplets were counted, the diameter of each droplet and average diameter of all droplets were calculated, and droplets were separated into size categories based on previously defined diameter ranges. Small droplets were designated as <2 µm and large lipid droplets were designated as >3 µm, unless otherwise stated. These outputs were exported into a CSV file. For each analysis, parameters of Image Analysis per Minute, number and diameter of lipid droplets and the CV (coefficient of variance) were calculated. The script used can be found in Supplementary Materials Figure S1.

### 2.7. Image Analysis—Cell Count

Images of cell nuclei stained with DAPI were run through a Python script for analysis. Using the OpenCV Python library (https://github.com/opencv/opencv (accessed on 9 September 2023)), images were loaded and converted to greyscale. Noise was reduced by adding Gaussian blur, and the image was converted to a binary image, separating the nuclei and the background by a previously defined threshold. Contours were detected, each contour was counted as a cell and its coordinates were marked by a rectangle. The image with the marked cells was exported as well as a CSV file with the cell count. The script used can be found in Supplementary Materials Figure S2.

### 2.8. RNA Extraction and cDNA Synthesis

After treatment, medium was removed, and the cells were harvested (Gene Elute Mammalian Total RNA miniprep kit; RTN70-1KT) according to the manufacturer’s instructions. Concentration and cleanliness levels of the RNA were determined using a nanodrop device (Thermo Scientific, Waltham, MA, USA) at 260/280 wavelengths. For cDNA synthesis we used the qScript cDNA Synthesis Kit (Quanta Biosciences, Beverly MA, USA). 1 μg of RNA was used in a PCR reaction using a PCR Lab Cycler (Sensoquest, Goettlingen, Germany).

### 2.9. Real-Time PCR Analysis

In order to test the expression level of the selected genes, we designed specific primers for each gene. Gene amplification reaction was performed using Quantabio PerfeCTa SYBR Green FastMix qPCR kit (Quanta Biosciences, Beverly, MA, USA) according to the manufacturer’s instructions (list of primers used in Table 1). Reaction efficiency and mRNA quantification in the sample was performed using the software LightCycler^®^ 96 (Roche, Basel, Switzerland), and the ΔΔCt method was used to calculate the relative expression of each gene. The expression of the selected genes was normalized using two genes as housekeeping genes (UXT, 18S). The expression levels of the tested genes were attributed to the geometric mean of the normalized genes.

### 2.10. Cell Viability Test

Cell viability was measured using the XTT Cell Proliferation Kit (Sigma-Aldrich, Rehovot, Israel). Cells (10,000 per well) were plated and grown for 24 h in a 96-well plate, with 100 microliters of medium added to each well. Upon reaching 100% confluence, the medium was removed and 100 µL of treatment medium containing 20% postbiotics and 80% growth medium was added to each well for the desired incubation period. Following incubation with the treatment, 30 µL of XTT reaction mix (containing 1:50 activation reagent to XTT reagent, *v*/*v*) was added to each well, and the plate was incubated for an additional 3 h. Lastly, samples were read at 630 nm using the BioTek Synergy H1 Multimode Reader (Winooski, VT, USA).

### 2.11. Mitochondrial Membrane Potential Determination

Cells were grown on glass coverslips in a 6-well plate with 2.5 mL of growth medium in each well. After reaching 100% confluence, treatment of 0.5 mM of H_2_O_2_ or 1 µg/mL of LPS in DMEM F/12 was added for another 4 h of incubation. Time frame was determined by preliminary study. Next, cells were washed with PBS and incubated with the JC-1 reagent (ENZO Life Sciences International, Plymouth Meeting, PA, USA) at a concentration of 153 µM for 10 min at 37 °C. JC-1 reagent is used as an indicator of mitochondria membrane potential. Following the incubation, cells were washed 3 times with PBS and the coverslips were mounted on slides with a fluorescent mounting medium (Dako, North America Inc., Carpinteria, CA, USA). Finally, cells were visualized with an Olympus BX40 fluorescence microscope equipped with an Olympus DP73 digital camera (Tokyo, Japan) using CellSens Entry software version 1.7.

### 2.12. Statistical Analysis

Statistical analysis was conducted using JMP software version 12.0.1 (SAS Institute, Cary, NC, USA), analyzing the results by one-way ANOVA. Comparisons were made by ANOVA followed by Tukey–Kramer HSD or Dunnett’s test. Significant probability was set to 0.05, and tendencies were reported at 0.05 < *p* ≤ 0.1. results are reported as mean ± standard error mean.

## 3. Results

### 3.1. MEC Response to Stress—Gene Expression and Mitochondria Potential after Exposure to LPS and H_2_O_2_

**Figure 1:**
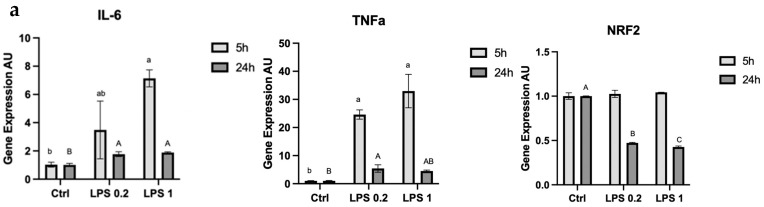

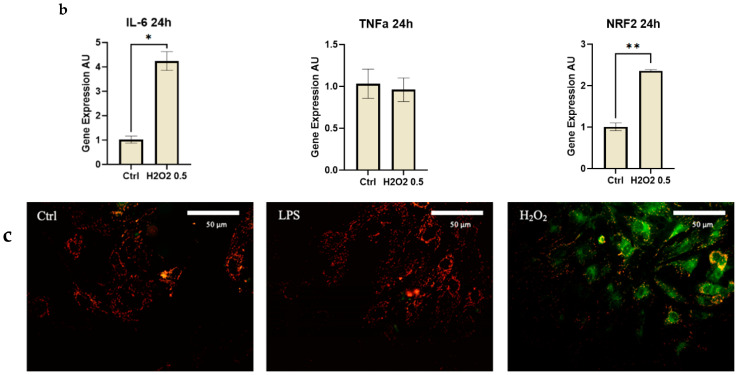
Gene expression levels of the proinflammatory cytokines IL-6 and TNFα and phase-2 protein NRF2 in MECs after (**a**) 5 or 24 h of exposure to 0.2 μg or 1 μg of LPS or (**b**) 24 h of exposure to 0.5 mM H_2_O_2_. Significant differences (*p* ≤ 0.05) are indicated with different letters or asterisks. Uppercase or lowercase letters are used for comparing results between treatments within the same time frame (5 or 24 h). (**c**) Representative images of MEC treated with 1 μg of LPS or 0.5 mM of H_2_O_2_ for 4 h compared with control, stained with JC-1, and visualized under a fluorescent microscope. Cells with impaired membrane potential appear green, whereas cells that maintain proper mitochondria membrane potential appear red.

In order to test MEC stress response, cells were exposed to LPS or H_2_O_2_ for 5 or 24 h, which were used to determine gene expression levels (Figure 1a,b). Expression levels of proinflammatory cytokines and phase 2 proteins had a different pattern between the two stressors and in terms of the time frame of gene expression modulation.

Overall, expression of proinflammatory cytokines peaked after 5 h of exposure to LPS and returned almost to basal levels after 24 h of exposure. Proinflammatory cytokine IL-6 showed a 3-fold and 7-fold increase after 5 h of exposure to 0.2 mg LPS and 1 mg LPS, respectively (*p* = 0.03). After 24 h of exposure to LPS, there was a 2-fold increase both after exposure to 0.2 and 1 mg/mL of LPS (*p* = 0.0109), and a 4-fold increase after 24 h exposure to H_2_O_2_ (*p* = 0.0487). TNFα, another proinflammatory cytokine, increased 25-fold and 35-fold after 5 h of exposure to 0.2 mg and 1 mg of LPS, respectively (*p* = 0.0027). The expression levels were elevated by 5-fold compared with control after 24 h of exposure to 0.2 mg and 1 mg of LPS (*p* = 0.0109), while there was no significant change with exposure to H_2_O_2_ for the same timeframe. The phase-2 protein NRF2 showed no significant change in expression after 5 h of exposure to LPS but showed a 2-fold decrease after 24 h of exposure to 0.2 mg and 1 mg of LPS, respectively (*p* < 0.0001), while 24 h of exposure to H_2_O_2_ led to a 2.5-fold increase (*p* = 0.0027).

The response to the two different stressors was also manifested in a change in mitochondria membrane potential (Figure 1c). MECs were exposed to LPS or H_2_O_2_ and the response of the mitochondria was completely different between treatments. While cells maintained proper mitochondria membrane potential after LPS treatment, the potential was significantly modulated by the H_2_O_2_ treatment.

**Figure 2:**
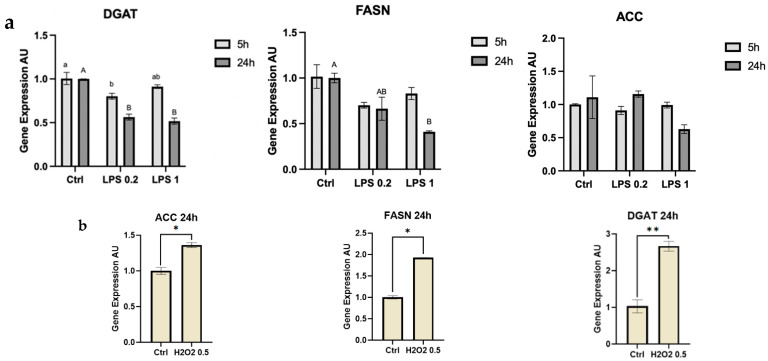
Gene expression levels of de novo fatty acid synthesis pathway genes, DGAT, ACC, and FASN in MECs after (**a**) 5 or 24 h of exposure to 0.2 or 1 μg of LPS or (**b**) 24 h of exposure to 0.5 mM H_2_O_2_. Significant differences (*p* ≤ 0.05) are indicated with different letters or asterisks. Uppercase or lowercase letters are used for comparing results between treatments within the same time frame (5 or 24 h).

De novo fatty acid synthesis-related genes were also measured and differences in expression levels after 24 h of treatment were recorded when LPS or H_2_O_2_ was used as a stressor (Figure 2). ACC did not show a significant change after exposure to LPS; however, it showed a 1.2-fold increase when exposed to H_2_O_2_ for 24 h (*p* = 0.0333). FASN and DGAT expression also increased after 24 h exposure to H_2_O_2_, increasing 2-fold and 2.5-fold, respectively (*p* = 0.0256, *p* = 0.0054), whereas FASN expression was reduced after 24 h of exposure to the higher dose of LPS by 0.5 fold (*p* = 0.0304), and similarly DGAT decreased 0.2-fold after 5 h exposure to the low LPS dose (*p* = 0.056) and decreased 0.5-fold after 24 h exposure to both doses of LPS, respectively (*p* = 0.0022). FASN expression did not show significant differences when exposed to LPS for 5 h but was reduced after 24 h of treatment.

### 3.2. Lipid Droplet Image Analysis

**Figure 3:**
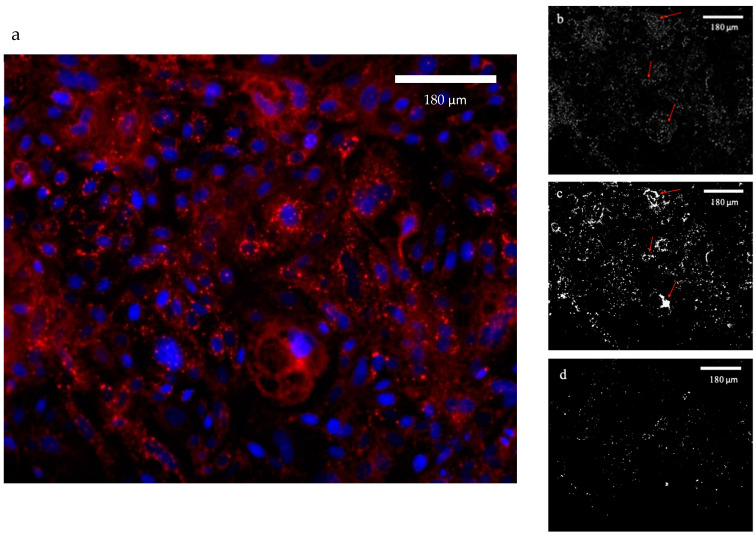
(**a**) Representative image of MECs nuclei stained with DAPI (blue) and lipid droplets stained with Nile red (red) and visualized under a fluorescence microscope and (**b**) the same image processed by a Python script. Large Lipid droplets that normally show clusters in the image and their size cannot be determined at high intensity are identified by the software and measured, indicated with arrows. Representative images processed by ImageJ software of MEC stained with Nile Red, under the conditions of (**c**) low contrast and (**d**) high contrast. Higher intensity results in clusters of large lipid droplets that are indistinguishable as separated droplets and therefore excluded by the software when size is recorded.

After exposure to oleic acid to induce triglyceride formation, and exposure to LPS for 24 h, cell nuclei and lipid droplets were stained with DAPI and Nile Red and mounted on glass slides. Lipid droplet size and quantity were evaluated under a microscope (see Figure 3a for a representative image). Initially, to determine the count and diameter of each lipid droplet, the software ImageJ was used. Images were converted to greyscale, and brightness and contrast were adjusted in order to eliminate background noise. To be able to recognize large lipid droplets, contrast needed to be decreased, reducing the ability to measure small lipid droplets that are usually stained with less intensity (see Figure 3d for representative images). To detect the smaller lipid droplets, contrast needed to be increased, causing the large lipid droplets to merge and become unmeasurable (see Figure 3c for representative image). When the same images were analyzed using a Python script, all images were adjusted equally and both large and small lipid droplets were clearly observed using the same contrast levels (see Figure 3b for a representative image).

The two methods were compared in terms of results and efficiency of image analysis. The manual analysis of images produced an average lipid droplet size of 1.77 with a co-efficiency variant of 0.74, while the automated analysis, probably due to higher sensitivity, generated an average lipid droplet size of 1.38 and a co-efficiency variant of 1.28 (Table 2). In terms of lipid droplet number per cell, the manual analysis generated an average of 4.57 with a co-efficiency variant of 22.07, and the automated analysis generated an average of 87.69 and a co-efficiency variant of 4.7 (Table 2). The coefficient of variance, which reflects the reproducibility of results, was similar between manual and automated analyses of lipid droplet size but was much higher in the manual analysis of the lipid droplet number.

### 3.3. MEC Response to Stress—Morphometric Features of Lipid Droplets

**Figure 4:**
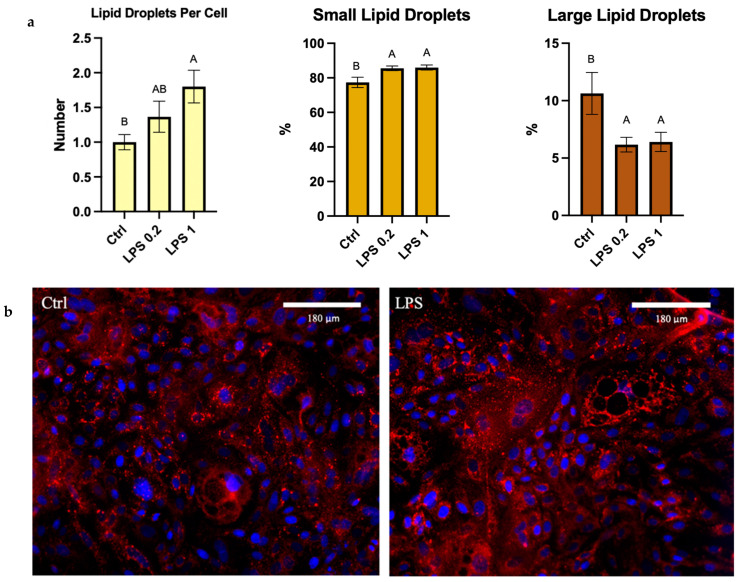
(**a**) Lipid droplet number and size distribution percentage out of total lipid droplets after 24 h of exposure to 1 μg of LPS. Significant differences (*p* ≤ 0.05) are indicated by different letters. (**b**) Representative images of MECs treated with 1 μg of LPS for 24 h compared with control (Ctrl). Lipid droplets stained with Nile Red (red) and Cell Nuclei stained with DAPI (blue).

The percentage of small lipid droplets significantly increased by 11% after exposure to LPS, in both concentrations (*p* = 0.0095). The percentage of large lipid droplets significantly decreased after exposure to both concentrations of LPS (*p* = 0.0295). The total number of lipid droplets per cell showed a 250-fold increase (*p* = 0.0236) (Figure 4a).

### 3.4. MEC Response to Exposure to Postbiotics from Bacillus subtilis Grown on Small and Large MFGs—Morphometric Features of Lipid Droplets

**Figure 5:**
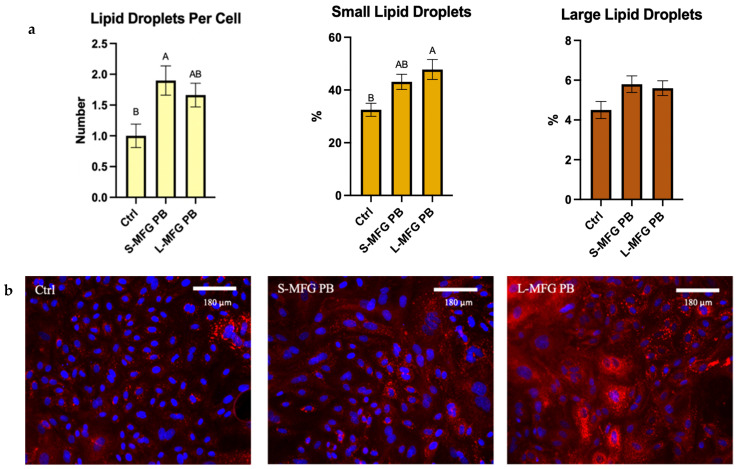
(**a**) Lipid droplet size distribution percentage out of total lipid droplets, after 24 h of exposure to postbiotics from bacteria grown on small or large MFGs (S-MFG PB and L-MFG PB, respectively). Significant differences (*p* ≤ 0.05) are indicated by different letters. (**b**) Representative images of MECs treated with postbiotics from *B. Subtilis* grown on small or large MFGs compared with control (Ctrl). Lipid droplets stained with Nile Red (red) and Cell Nuclei stained with DAPI (blue).

The percentage of small lipid droplets significantly increased by 16% and 11% after exposure to postbiotics from small and large MFGs, respectively (*p* = 0.0041). Exposure to postbiotics from small and large MFGs led to a 1.5-fold and 2-fold increase in lipid droplet number per cell, respectively (*p* = 0.014) (Figure 5a).

### 3.5. MEC Response to Postbiotics from Bacillus subtilis Incubated with Small or Large MFGs—Gene Expression

**Figure 6:**
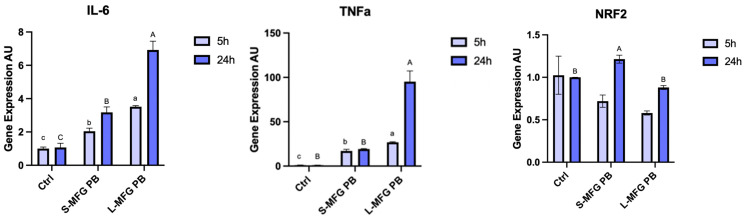

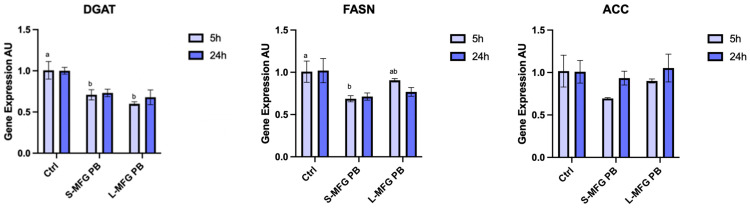
Gene expression levels of proinflammatory cytokines IL-6 and TNFα, the phase-2 protein NRF2, and of de novo fat synthesis-related genes in MECs treated for 5 or 24 h with postbiotics from *B. Subtilis* grown on small or large MFGs (S-MFG PB and L-MFG PB, respectively). Significant differences (*p* ≤ 0.05) are indicated with different letters. Uppercase or lowercase letters are used for comparing results between treatments within the same time frame (5 or 24 h).

In this part of the experiment, postbiotics were collected from bacteria grown on small or large MFGs as a substrate and used as a conditioning medium for MECs. The response of the cells was recorded after 5 and 24 h (Figure 6).

Proinflammatory cytokines were elevated after 24 h of exposure, with a more significant response to postbiotics of bacteria grown on large MFGs. More specifically, IL-6 showed a 2-fold and 3-fold increase after exposure to postbiotics from small and large MFGs for 5 h, respectively (*p* = 0.0002). IL-6 expression increased 3-fold and 7-fold after 24 h of exposure to postbiotics collected from bacteria grown on small or large MFGs, respectively (*p* = 0.0001). TNFα expression also increased when exposed to postbiotics from small and large MFGs, increasing 15-fold and 25-fold after 5 h, respectively (*p* = 0.0001). After 24 h TNFα expression increased 25-fold and 100-fold when cells were exposed to postbiotics from bacteria grown on small and large MFGs, respectively (*p* = 0.0002). The phase-2 protein NRF2 increased 1.2-fold in MECs exposed to postbiotics from small MFGs for 24 h (*p* = 0.0029).

Gene-encoding enzymes involved in de novo lipid synthesis showed expression patterns similar to those found when MECs were exposed to LPS and were not significantly different between postbiotics originating from bacteria grown on large or small MFGs. DGAT expression decreased when MECs were exposed to postbiotics from both small and large MFGs, 0.25-fold and 0.5-fold, respectively (*p* = 0.017). FASN behaved similarly, decreasing 0.5-fold and 0.25-fold after 5 h of exposure to postbiotics from small MFGs and large MFGs, respectively (*p* = 0.0087). Similarly to the exposure to LPS, ACC did not show a significant difference in expression (*p* = 0.1053) upon exposure to postbiotics.

### 3.6. MEC Response to Exposure to Postbiotics from E. coli Grown on Small and Large MFGs—Morphometric Features of Lipid Droplets

**Figure 7:**
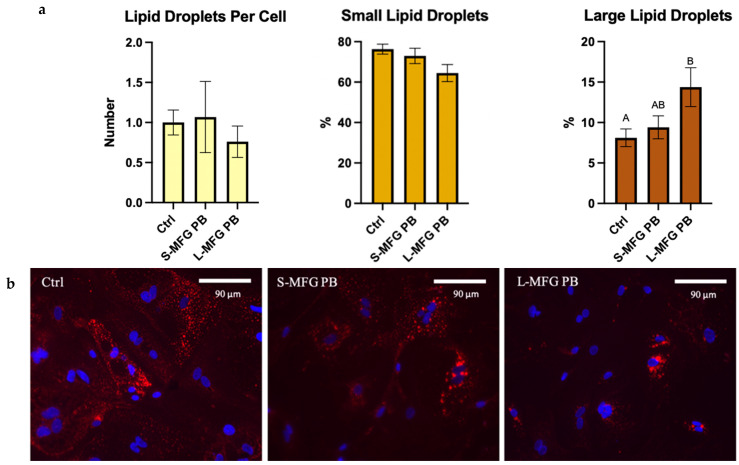
(**a**) Lipid droplet size distribution percentage out of total lipid droplets, after 24 h of exposure to postbiotics from *E. coli* grown on small or large MFGs (S-MFG PB and L-MFG PB, respectively). Significant differences (*p* ≤ 0.05) are indicated with different letters. (**b**) Representative images of MEC treated with postbiotics from *E. coli* grown of small or large MFGs compared with control (Ctrl). Lipid droplets stained with Nile Red (red) and Cell Nuclei stained with DAPI (blue).

Overall, lipid droplet morphometric features did not resemble those of MECs under stress as the percentage of small lipid droplets did not significantly change (*p* = 0.063), whereas that of large lipid droplets significantly increased after exposure to postbiotics from large MFGs (*p* = 0.0325). The number of lipid droplets per cell also did not change significantly (*p* = 0.7419) (Figure 7a).

### 3.7. MEC Response to Postbiotics from E. coli Grown on Small and Large MFGs—Gene Expression

**Figure 8:**
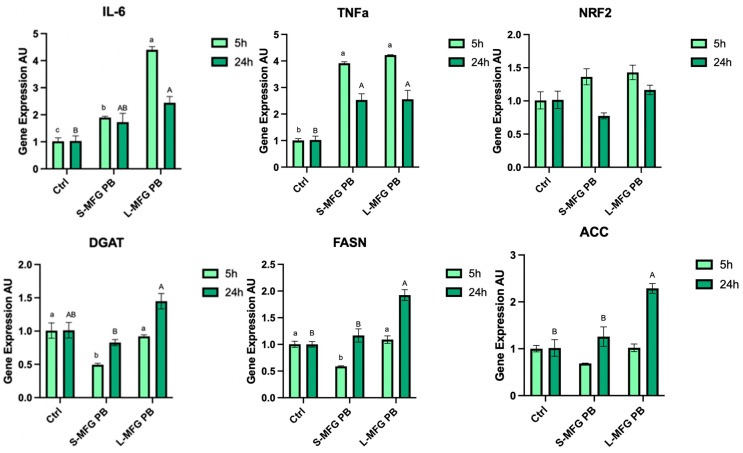
Gene expression levels of proinflammatory cytokines IL-6 and TNFα, of the phase-2 protein NRF2, and of de novo fat synthesis-related genes in MECs after exposure of 5 or 24 h to postbiotics from *E. coli* grown on small or large MFGs (S-MFG PB and L-MFG PB, respectively). Significant differences (*p* ≤ 0.05) are indicated with different letters. Uppercase or lowercase letters are used for comparing results between treatments within the same time frame (5 or 24 h).

Overall, expression of proinflammatory cytokines in MECs was elevated in response to postbiotics, with a greater impact after exposure to postbiotics collected from *E. coli* grown on large compared with small MFGs (Figure 8). The expression of proinflammatory cytokine IL-6 increased after 5 and 24 h of exposure to *E. coli* postbiotics from both small and large MFGs (*p* < 0.0001, *p* = 0.0219). Postbiotics from small MFGs led to a 2-fold increase after 5 and after 24 h, and postbiotics from large MFGs led to a 5-fold and 2.5-fold increase after 5 and 24 h of exposure, respectively. TNFα expression increased to a similar extent when MECs were exposed to postbiotics from bacteria grown on small and large MFG with a 4-fold increase after 5 (*p* < 0.0001, *p* = 0.0077), and a 2.5-fold increase after 24 h (*p* < 0.0001, *p* = 0.0077). The phase-2 protein NRF2 did not show significant changes after exposure to either of the postbiotics, after 5 h or 24 h (*p* = 0.1372, *p* = 0.095) (Figure 8).

All de novo fat synthesis-related genes that were measured increased after 24 h of exposure to postbiotics from large MFGs. DGAT increased 1.5-fold (*p* = 0.0188) FASN increased 2-fold (*p* = 0.0113) and ACC increased 2.5-fold (*p* = 0.0236). DGAT and FASN both showed a 0.5-fold decrease after 5 h of exposure to postbiotics from small MFGs (*p* = 0.0078, *p* = 0.0062).

### 3.8. MEC Response to Exposure to Postbiotics from Bacillus subtilis and E. coli Grown on Small and Large MFGs—Cell Viability Test

**Figure 9:**
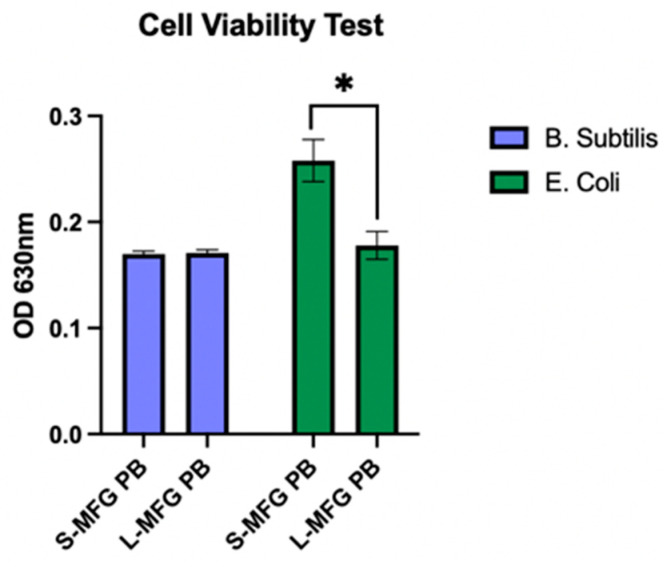
MEC viability after 5 h of exposure to postbiotics from *B. subtilis* or *E. coli* grown on either small or large MFGs (S-MFG PB and L-MFG PB, respectively). Asterisks indicate significant differences (*p* ≤ 0.05).

Cell viability was tested using an XTT cell proliferation kit, after 5 h of exposure to postbiotics collected from *B. subtilis* and *E. coli* grown on either small or large MFGs (Figure 9). We found that the viability of cells exposed to *E. coli* postbiotics from large MFGs was lower than that of small MFGs (*p* = 0.05), while there was no significant difference in cell viability after exposure to *B. subtilis* postbiotics from small and large MFGs (*p* = 0.749).

## 4. Discussion

The aim of the present study was to assess whether the stress response in MECs includes the modulation of morphometric features of intracellular lipid droplets. To characterize the interaction between stress and lipid droplet size, we used a computational approach, which allowed us to characterize the stress response of the lactating dam through the lipid output of the gland. Once the interaction between lipid droplet features and stress was identified, we implemented it on MECs exposed to postbiotics from bacteria that naturally populate the milk cavities of dairy cow udders. Two conditions were used to change the postbiotics composition: (i) the postbiotic origin—commensal or pathogenic bacteria, and (ii) the MFG size used as a substrate by the bacteria.

To study whether MFG size can be used as an indicator for stress, we used common exogenous stressors like LPS and H_2_O_2_ and studied their implication on morphometric features of the MFG precursors, the intracellular lipid droplets. The stress response was confirmed by elevated expression of the proinflammatory gene IL-6. TNFα, another proinflammatory factor, differed in response between LPS and H_2_O_2_, as did the phase-2 protein NRF2; both IL-6 and TNFα expression peaked after 5 h of exposure to LPS, with a moderate response after 24 h. H_2_O_2_ on the other hand showed no TNFα response after 24 h (Figure 1). These findings coincide with previous studies that used LPS in rats to demonstrate an acute response of IL-6 expression that subsided within 2 h, with a more delayed response lasting up to 10 h seen in TNFα [24]. These results suggest that the response to different stressors can follow the same proinflammatory molecular changes, with distinguished effects on energy and lipid metabolism genes both in the short-immediate time range and in the long term (after 24 h).

Our results show that mitochondrial membrane potential was impaired and levels of NRF2 expression were elevated following the H_2_O_2_ treatment but not the LPS treatment (Figure 1). This could be attributed to the fact that LPS stimulates expression of proinflammatory cytokines IL-6 and TNFα [25,26] by binding to Toll-Like Receptor 4 (TLR4), whereas H_2_O_2_ is rapidly broken down into reactive oxygen species (ROS). Elevated ROS levels induce the release of the NRF2 protein from its chaperone, allowing it to activate the phase 2 antioxidant response [27].

There is a tight biochemical and molecular interaction between mitochondria activity and the lipogenic gene network [28,29]. Mitochondrial activity is closely related to lipid metabolism, as the precursor of fatty acids is synthesized within the mitochondria cytoplasm, with pyruvate being converted into acetyl Coenzyme A as part of the oxidative phosphorylation process [30]. Previous studies in the liver have shown that oxidative stress leads to increased production and accumulation of lipid droplets [31], correlating to our findings of increased expression of lipogenic genes such as DGAT, ACC, and FASN [32] after exposure to H_2_O_2_. Moreover, in HepG2 cells, H_2_O_2_ was shown to increase the expression of the transcriptional factor Sterol Regulatory Binding Element 1c (SREBP1c), and consequently increase its downstream lipogenic genes, resulting in lipid accumulation [33]. These findings are consistent with the increase in DGAT, ACC, and FASN that we saw in MECs exposed to H_2_O_2_ (Figure 2).

On the other hand, we found that exposure to LPS leads to a decreased expression of both FASN and DGAT. The fact that FASN expression was decreased due to LPS (Figure 2) is in accordance with previous studies that showed that after inoculation with the bacteria *Streptococcus uberis*, FASN, along with many other lipogenesis-related genes, was down-regulated [34]. A similar decrease in DGAT and FASN and a myriad of lipogenic gene expressions under stress was shown in dairy cows with elevated plasma LPS concentrations induced by the consumption of a high-concentrate diet [35]. In accordance with our findings, it was previously suggested that LPS reduces the expression of SREBP1 and its downstream genes, which include DGAT, FASN, and ACC [36].

After confirming the proinflammatory response of MECs to both LPS and H_2_O_2_, we sought to study the impact on the size and number of intracellular lipid droplets in these cells. The interaction between stress and lipid droplet features was demonstrated in lipogenic tissues like liver pathologies [15] but was not yet demonstrated in MECs. Moreover, lipid droplets in MECs are the precursors of secreted MFGs. Therefore changes in lipid droplet size can impact milk lipid composition including fatty acids, polar lipids, and the content of MFGM, and hence milk bioactivity [22,37,38,39].

Using the common software to run lipid droplet size and number diagnoses, we found that images must be adjusted in a way that will either impair our ability to quantify large lipid droplets or the capacity to determine the size of small lipid droplets. This technical limitation stems from the fact that large lipid droplets are stained with higher intensities compared with small ones. This can be attributed to the greater hydrophobicity of larger droplets, which absorb more of the hydrophobic dye. Therefore, the analysis of each image was biased toward either small or large droplets, depending on imaging intensity used.

To overcome these limitations, we used a Python script which allowed us to adjust each image equally and perform a single run to capture a much wider spectrum of lipid droplet sizes (Figure 3). The results show that under stress, lipid droplet number is increased, and size is decreased (Figure 4). This is in accordance with previous studies showing that diseases such as non-alcoholic fatty liver disease (NAFLD) lead to the accumulation of small lipid droplets in the liver [40]. The fact that stress is associated with the formation of small lipid droplets was also demonstrated in muscle cells exposed to energy deprivation [17]. In the mammary gland, however, it was previously demonstrated that mastitis leads to the secretion of larger MFGs [41], suggesting that different types of stressors may elicit a different response, as we saw in the expression level of lipogenic genes after exposure to LPS and H_2_O_2_. In addition, the involvement of other cell types, like immune cells in the in vivo settings as in the case of mastitis, may also contribute to the different phenotype.

Finally, we hypothesized that different bacteria strains could alter the MFG phenotype. Hence, we used the phenotype of lipid droplet size and number to assess the stressogenic level of bacteria secretome on MEC. Previous studies showed that the MFG size changes the growth trajectories of *Bacillus subtilis* and its capacity to form a biofilm [42]. In order to shift from proliferation mode to biofilm formation mode, the bacteria activate a different set of genes that affect how it metabolizes nutrients and what the chemical profile of its secretions is. After incubation of the bacteria with large MFGs, there is a reduction in metabolites relating to pathways that induce biofilm formation such as UDP-Galactose. Metabolites relating to the Citric Acid cycle such as a-ketoglutaric acid, citric acid, and succinic acid increase after 24 h of incubation with small MFGs, indicating an increase in proliferation. In the present study, the secretome was collected from two types of bacteria that are part of the dairy cows’ udder microbiome—*Bacillus subtilis* [43] and *E. coli* [44]. Since these bacteria are part of the mammary gland microbiome, their secreted metabolites can directly affect the epithelial lining of the mammary gland, and hence MECs. Therefore, in physiological settings, where bacteria populate the body cavities of the udder (i.e., milk collecting ducts), the host’s MEC will be exposed to the secretome of the bacteria, which might induce a proinflammatory or a stress response and affect productivity and milk quality. Milk lipid content and composition may respond to stress, as was previously reported by Liu et al., who showed that cows under heat stress produced milk with an altered fatty acid composition [45].

We found that the secretome of *B. subtilis* led to an increase in the percentage of small lipid droplets in the cells, regardless of the size of MFGs used as a substrate to produce the postbiotics (Figure 5). This was in accordance with the stress response recorded through inflammatory gene expression, although exposure to postbiotics from bacteria grown on large MFGs elicited a stronger stress response than the small MFGs postbiotics (Figure 6). This difference in the extent of the response to stress was not reflected in the phenotype of lipid droplet size or number, which was similar in both postbiotic treatments. The smaller lipid droplets may also be attributed to the decreased expression of lipogenic genes such as DGAT and FASN (Figure 6). This response is similar to the response of MECs to LPS.

The changes in lipogenic gene expression shown may indicate changes in the lipogenesis and lipolysis processes occurring in the cells in response to stress, leading to varying phenotypes in terms of lipid droplet morphometric features.

The *E. coli* secretome elicited a different lipid droplet size response in MECs, depending on the MFG size used as a substrate for the bacteria (Figure 7). In terms of proinflammatory gene expression, both treatments elevated gene expression of the cytokines, with higher expression in MECs exposed to postbiotics from bacteria grown on large MFGs. In addition, after exposure to postbiotics from *E. coli* grown on large MFGs, there was an increase in large lipid droplet percentage and a decrease in overall lipid droplet count. This was the only treatment that increased the expression of proinflammatory genes along with increased lipid droplet size (Figure 8). The fact that lipid droplet size is uncoupled from the proinflammatory response is also manifested by differences in the response of lipogenic genes that were reduced after 5 h only by postbiotics from small MFGs, whereas after 24 h, they were elevated only by postbiotics from the large MFGs.

It was previously shown that cell apoptosis can lead to the generation of large lipid droplets [46]. This is in agreement with our findings that postbiotics from *E. coli* grown on large MFGs lead to a viability decrease and a lipid droplet size increase in MECs (Figure 9). This result is in contrast with *B. subtilis* postbiotics, which showed similar viability levels between different postbiotic treatments.

## 5. Conclusions

Taken together, the various aspects of lipid production and metabolism may be affected by stress and/or other factors that exist in bacterial postbiotics, and therefore lead to morphological changes in lipid droplets synthesized by MECs. Postbiotics are secreted natively in the mammary gland and can therefore act as modulators of mammary gland lipogenic activity. Exposure to different bacteria leads to varying responses that are expressed in the morphology of synthesized lipid droplets and their composition. This could hint at a potential underlying reason for the wide size range of lipid droplets secreted by the mammary gland as MFGs, such as protection against pathogenic bacteria, or providing a competitive advantage to commensal bacteria over other strains present in the gland.

## Figures and Tables

**Table 1:** List of Primers.

Gene Name	Accession Num.	Forward Sequence (5′-3′)	Reverse Sequence (5′-3′)
18S	NC_037354.1	CGGCTACCACATCCAAGGAA	GGGCCCCGAAAGAGTCCTG
UXT	NM_001037471.2	TGTGGCCCTTGGATATGGTT	GGTTGTCGCTGAGCTCTGTG
IL-6	NM_173923.2	GCTGAATCTTCCAAAAATGGAGG	GCTTCAGGATCTGGATCAGTG
TNFα	NM_173966.3	TCTTCTCAAGCCTCAAGTAACAAGT	CCATGAGGGCATTGGCATAC
NRE2L2 (NRF2)	NM_001011678.2	AGGACATGGATTTGATTGAC	TACCTGGGAGTAGTTGGCA
DGAT	NM_174693.2	CGACTCCTGGAGATGCTGTT	ATGCGGGAGTAGTCCATGTC
FASN	NM_001012669.1	GCATCGCTGGCTACTCCTAC	GTGTAGGCCATCACGAAGGT
ACC	NM_174224.2	AGCTGAATTTTCGCAGCAAT	GGTTTTCTCCCCAGGAAAAG

**Table 2:** Manual vs. Automated analyses.

Analysis	Lipid Droplet Size	SD	CV	Lipid Droplet Number ^a^	SD	CV	Analysis Per Min
Manual	1.77	0.013	0.74	4.57	1.01	22.07	<1 image
Automated	1.38	0.018	1.28	87.69	4.13	4.70	30 images

^a^ average lipid droplet number per cell.

## Data Availability

The original contributions presented in the study are included in the article/Supplementary Material. Script repositories are available in the following links: https://github.com/scikit-image/scikit-image, https://github.com/opencv/opencv. Further inquiries can be directed to the corresponding author.

## Supplementary Materials

The following supporting information can be downloaded at: https://www.mdpi.com/article/10.3390/foods13152429/s1, including Figure S1. a python script for lipid droplet analysis and Figure S2. a python script for cell count.
