# Supplementary material for: The Stressogenic Impact of Bacterial Secretomes Is Modulated by the Size of the Milk Fat Globule Used as a Substrate
Source: Foods. 2024 Aug 1;13(15):2429. doi: 10.3390/foods13152429 (PMC11312077; doi:10.3390/foods13152429)
Supplement: Supplementary file 1 [file foods-13-02429-s001.zip › foods-3111216-supplementary.pdf]

Supplementary Material

1. Python Script – Lipid Droplet Analysis

```
import os
import datetime
import numpy as np
from skimage import io, color, exposure, feature
import cv2
from PIL import Image
import multiprocessing


input_folder = "/ImagesFolderPath/Images/"
output_folder = os.path.join(os.path.dirname(input_folder),
"Image_Outputs")
timestamp = datetime.datetime.now().strftime("%Y%m%d%H%M%S")
output_folder_timestamped = os.path.join(output_folder, timestamp)
os.makedirs(output_folder_timestamped, exist_ok=True)

def process_image(image_path):
    img = io.imread(image_path)
    img = exposure.rescale_intensity(img, in_range='image',
out_range=(0, 1))
    img = exposure.rescale_intensity(img, in_range=(0.2, 0.8),
out_range=(0, 1))

    img_gray = color.rgb2gray(img)
    img_contrast = exposure.equalize_adapthist(img_gray,
clip_limit=0.03)

    blobs = feature.blob_log(img_contrast, max_sigma=5,
threshold=0.01)
    black_background = np.zeros_like(img_gray)

    particle_count = 0
    sum_diameter = 0.0

    for blob in blobs:
        y, x, r = blob
        if r > 2:
            cv2.circle(black_background, (int(x), int(y)), int(r),
(1, 1, 1), -1)
            particle_count += 1
            sum_diameter += 2 * r

    output_path = os.path.join(output_folder_timestamped,
os.path.basename(image_path))
    Image.fromarray((black_background *
255).astype(np.uint8)).save(output_path, format="PNG")

    return os.path.basename(image_path), particle_count,
sum_diameter

if __name__ == '__main__':
    num_processes = os.cpu_count()
```

```
    with multiprocessing.Pool(processes=num_processes) as pool:
        image_paths = [os.path.join(input_folder, filename) for
filename in os.listdir(input_folder) if
                       filename.endswith(".jpg") or
filename.endswith(".png")]

        results = pool.imap_unordered(process_image, image_paths)

        for filename, particle_count, diameter_sum in results:
            if particle_count > 0:
                average_diameter = diameter_sum / particle_count
            else:
                average_diameter = 0.0
            print(f"Image: {filename}, Particles: {particle_count},
Average Diameter: {average_diameter:.2f} pixels")

    print("Image processing completed and saved in:",
output_folder_timestamped)
```

2. Python Script – Cell Count

```
3. import cv2
   import numpy as np
   import os
   import openpyxl

   def count_cells(image_path):
       image = cv2.imread(image_path)

       if image is None:
           print(f"Error: Unable to load the image from
   '{image_path}'")
           return 0

       gray = cv2.cvtColor(image, cv2.COLOR_BGR2GRAY)
       blurred = cv2.GaussianBlur(gray, (5, 5), 0)
       _, thresh = cv2.threshold(blurred, 0, 255, cv2.THRESH_BINARY
   + cv2.THRESH_OTSU)
       contours, _ = cv2.findContours(thresh, cv2.RETR_EXTERNAL,
   cv2.CHAIN_APPROX_SIMPLE)
       marked_image = image.copy()

       cell_count = 0
       for contour in contours:
           area = cv2.contourArea(contour)

           if 100 < area < 25000:
               x, y, w, h = cv2.boundingRect(contour)
               cv2.rectangle(marked_image, (x, y), (x + w, y + h),
   (0, 255, 0), 2)
               cell_count += 1

       return cell_count, marked_image


   def process_images_in_folder(folder_path):
```

```
    results = []
    excel_filename = "cell_counts.xlsx"
    output_folder = os.path.join(folder_path, "cell_outputs")
    os.makedirs(output_folder, exist_ok=True)

    wb = openpyxl.Workbook()
    ws = wb.active
    ws.append(["Image Name", "Cell Count"])

    for filename in os.listdir(folder_path):
        if filename.endswith(('.jpg', '.jpeg', '.png', '.bmp')):
            image_path = os.path.join(folder_path, filename)
            cell_count, marked_image = count_cells(image_path)
            results.append((filename, cell_count))
            marked_image_path = os.path.join(output_folder,
f"marked_{filename}")
            cv2.imwrite(marked_image_path, marked_image)
            ws.append([filename, cell_count])

    wb.save(os.path.join(output_folder, excel_filename))
    return results

if __name__ == "__main__":
    folder_path = "/ImagesFolderPath/Images/"
    if not os.path.exists(folder_path):
        print(f"Error: Folder '{folder_path}' does not exist.")
    else:
        results = process_images_in_folder(folder_path)
        print("Analysis complete. Results saved to 'cell_outputs'
folder.")
```
